# Health-related quality of life among multimorbid patients using the EQ-5D-5L value set for India

**DOI:** 10.3389/fpubh.2025.1612512

**Published:** 2025-09-18

**Authors:** Krushna Chandra Sahoo, Abhinav Sinha, Girish Chandra Dash, Rakesh Kumar Sahoo, Debdutta Bhattacharya, Purna Chandra Dash, Lalatendu Mohanty, Pranab Mahapatra, Kavitha Rajsekhar, Sanghamitra Pati

**Affiliations:** ^1^Health Technology Assessment India, Department of Health Research, Ministry of Health & Family Welfare, New Delhi, India; ^2^Health Technology Assessment India, Regional Resource Hub, ICMR-Regional Medical Research Centre, Bhubaneswar, India; ^3^Department of General Medicine, S.C.B. Medical College & Hospital, Cuttack, India; ^4^Department of Medicine, Kalinga Institute of Medical Sciences, Bhubaneswar, India; ^5^Department of Psychiatry, Kalinga Institute of Medical Sciences, Bhubaneswar, India

**Keywords:** multimorbidity, quality of life, EQ-5D-5L, utility score, Health Technology Assessment, multiple long-term conditions

## Abstract

Multimorbidity presents major challenges to healthcare systems worldwide. Assessing health-related quality-of-life (HRQoL) in multimorbid patients is essential for understanding the overall impact of the patient’s health conditions on wellbeing and the complexities of patient management. This study assessed the HRQoL of multimorbid patients in India using the EQ-5D-5L value set. This observational study included 906 patients from tertiary healthcare facilities in Odisha, India, and used consecutive time-based sampling methods, conducted from January to April 2023. The study examined the relationship between HRQoL measures and utility scores using ordinary least squares regression and multiple regression analysis. The results showed that mean utility scores decreased as the number of health conditions increased, with scores of 0.677 for one condition, 0.577 for two conditions, 0.354 for three conditions, and 0.098 for four or more conditions. Combining stroke/paralysis with other health issues resulted in negative utility ratings. The findings showed that younger age (*p* = 0.003), urban residence (*p* = 0.027), higher education (*p* = 0.018), being married (*p* = 0.006), engaging in physical activity (*p* = 0.001), and having fewer health conditions were independently associated with higher utility scores. The study highlights the correlation between multimorbidity and HRQoL in older adults, highlighting implications for healthcare systems and clinical and policy decisions for multimorbid patients.

## Introduction

Multimorbidity, characterised by the simultaneous presence of two or more chronic health conditions in an individual, has profound implications for the health-related quality of life (HRQoL) (1–3). The intricate interplay of multiple health issues often presents formidable challenges in the management of daily activities, exerting impacts on both physical and mental wellbeing (4). Individuals grappling with multimorbidity may encounter limitations in functional capacity, leading to an overall decline in their HRQoL (5). Managing the consequences of multiple health conditions, scheduling multiple medications, and navigating intricate healthcare systems can lead to increased stress levels and reduced satisfaction in daily activities. Furthermore, the cumulative effects of multiple chronic conditions may intensify symptoms, diminish resilience, and impede one’s ability to participate in social activities (6, 7). Understanding and appropriately addressing the various consequences of multimorbidity, specifically its influence on HRQoL, is essential for developing comprehensive healthcare strategies to enhance the overall wellbeing of individuals coping with the complexities of managing multiple chronic health conditions.

Currently, there is a growing emphasis on understanding diseases and healthcare from the perspective of patients, emphasising patient-reported outcome measures (8). Therefore, the importance of HRQoL data for patient-centred care has increased, offering a valuable understanding of individuals’ subjective experiences regarding their health and wellbeing (9). Furthermore, evaluating HRQoL enables healthcare practitioners to assess the influence of medical treatments and interventions on the overall wellbeing of patients (10, 11). The HRQoL data are crucial for informing healthcare decision-making processes by providing a comprehensive view of the physical, mental, and social dimensions of health (12–14). Additionally, previous studies have underscored the critical role of nurses’ awareness, attitude, and self-efficacy in ensuring patient safety (9–11). Collectively, these factors contribute to creating a safer healthcare environment, ultimately improving health outcomes and HRQoL for patients, including those with multimorbidity. This information is crucial for prioritising interventions, allocating resources effectively, and shaping policies to meet diverse patient populations’ needs.

The literature indicates that HRQoL among multimorbid patients is generally lower compared to individuals with single chronic conditions due to the cumulative burden of multiple conditions and complexity in the management of multiple conditions (8–12). Despite recognising the impact of multimorbidity on HRQoL, existing research in the Indian context is limited in exploring how specific combinations of chronic conditions influence HRQoL scores derived from the EQ-5D-5L. Additionally, there is a lack of comprehensive data on the prevalence and severity of multimorbidity across different demographic and socioeconomic groups in India (13–15). The EQ-5D-5L, a widely used instrument for measuring HRQoL, has been validated, utilising the EQ-5D-5L value set for India (16), highlighting the need for integrated healthcare approaches to improve the overall quality of life among multimorbid patients. Addressing these gaps is essential for designing targeted interventions and informing health policy, underscoring the need for further studies to elucidate the nuanced relationship between multimorbidity patterns and quality of life outcomes. However, there is a research gap in understanding how specific patterns of multimorbidity affect HRQoL among Indian patients, and therefore, further study is essential to fill that gap by assessing the relationship between multimorbidity combinations and EQ-5D-5L-based quality of life scores in the Indian context.

Multimorbidity can significantly affect patients’ relationships, lead to increased financial burdens due to ongoing healthcare costs, and pose challenges in accessing appropriate medical services, especially in resource-limited settings. Given India’s rising burden of chronic diseases and the growing prevalence of multimorbidity, understanding its impact on HRQoL is particularly relevant to inform targeted healthcare strategies and improve patient outcomes in the Indian context (3, 4). Addressing these social dimensions is essential for a comprehensive understanding of the true burden of multimorbidity and for developing holistic interventions to improve overall wellbeing. The HRQoL constitutes a crucial element in economic modelling, specifically within decision trees and Markov models. The EQ-5D-5L stands out as a widely employed generic tool for assessing and appraising health status (15). It furnishes a straightforward descriptive profile and a single index for health status, applicable in both clinical and economic evaluations of healthcare, as well as in population health surveys. Despite the prevalent use of EQ-5D-5L in health technology assessment in India, the country lacked a dedicated EQ-5D-5L value set. Recently, Jyani et al. (16) addressed this gap by providing utility scores for each health state within the Indian context (16). The hypothesis proposed that people with multiple chronic conditions may face more restrictions in physical and functional activities, lowering their overall HRQoL. Furthermore, it was hypothesised that the HRQoL for older people with multimorbidity would be lower than for their younger counterparts. Therefore, in this study, we assessed the self-perceived HRQoL of multimorbid patients in India using the EQ-5D-5L value set—utility score for each health state. This will provide information for health technology assessment as well as additional guidelines for future clinical and policy decision-making for the management of multimorbid patients in India.

## Methods

### Study design, participants, and setting

An observational study was conducted among patients aged ≥18 years who visited the medicine outpatient department (OPD) at two tertiary healthcare and teaching hospitals in the state of Odisha, India. We have included patients aged 18 years and above attending the medicine OPD; first-time attendees on the designated sampling days, and patients providing informed consent to participate in the study. We have excluded the patients unable to participate due to insufficient cognition or debilitating conditions, inpatients, repeat visitors previously interviewed in the current study, and patients unwilling to participate or who refused consent. The participating facilities were the Kalinga Institute of Medical Science, Bhubaneswar (private), and SCB Medical College in Cuttack (public), representing both private and public healthcare sectors.

### Sample size and sampling frame

The complex multimorbidity prevalence in India was 34.5% (17). The calculation of the sample size was predicated on specific parameters, including the population size (*N*) with a finite population correction factor, a hypothesized 34.5% frequency of the outcome factor (p), 95% confidence limits (*Z*1 − *α*/2 = 1.96), precision (*d* = 0.1), and the design effect for cluster surveys (DEFF = 1). The sample size (*n*) was determined using the equation: *n* = [DEFF*Np(1-p)]/ [*d*^2^/*Z*^2^_1-*α*/2_*(*N*-1) + p*(1-p)]. The resulting required sample size was determined to be 348 at each facility.

We used consecutive time-based sampling methods. Through discussions with healthcare professionals at each institution, it was determined that approximately 70–100 adult patients visited the medicine OPD daily. Among them, a minimum of 25 were first-time attendees. Consequently, the decision was made to include all first-time visitors on every alternate working day, totalling around 12 days per month. This approach aimed to enrol at least 10 patients per day over 4 months, to reach a sample size of 480 patients at each facility. We were able to collect 403 responses from private and 503 from public health facilities. Data collection took place from January to April 2023.

### Study variables and data collection procedure

We used a pre-validated structured Multimorbidity Assessment Questionnaire for Primary Care (MAQ-PC) to collect data. This instrument has a high internal consistency (Cronbach’s alpha: 0.69), interrater reliability (Cohen’s Kappa: 0.78–1), and test–retest reliability (ICC: 0.970–0.741) (18). A tablet-based Open Data Kit (ODK) format was designed to efficiently capture information encompassing socio-demographic details, multimorbidity assessment, and EQ-5D-5L. The multimorbidity assessment section of the MAQ-PC included self-reported doctor-diagnosed chronic conditions such as diabetes, musculoskeletal disorders, acid peptic disease, disabilities, visual and hearing impairments, arthritis, neurological conditions, chronic lung disease, cancer, tumour, thyroid diseases, heart disease and stroke, chronic kidney diseases, hypertension, and certain chronic infectious diseases like tuberculosis, HIV, and filariasis.

To enhance data validity, these self-reported conditions were cross-validated using prescription records obtained from patient pharmacy data. A subset of the data was used to construct a misclassification matrix comparing self-reported diagnoses with prescription-based diagnoses. The sensitivity, specificity, and predictive values indicated high concordance (substantial agreement, kappa = 0.78). Conditions were selected based on their clinical relevance, prevalence in the population, and data availability. Conditions with very low prevalence or unreliable self-reporting were excluded to improve data quality and analytical robustness.

The EQ-5D-5L, a commonly employed health-related quality-of-life tool, entails a thorough evaluation of an individual’s health status across five dimensions: mobility, self-care, usual activities, pain/discomfort, and anxiety/depression (16, 19). Each dimension encompasses five response levels, providing a more detailed assessment. For each dimension, a single question is posed, with five options on a Likert scale ranging from 1 (indicating no problems) to 5 (signifying an inability to perform the activity or experiencing extreme pain, anxiety, or depression). The EQ5D tool was evaluated the validity, reliability, and responsiveness of in regional language (Odia) version (20).

Consenting participants were interviewed following consultation with the relevant physician in the medicine department, ensuring minimal disruption to the hospital’s system. Exit interviews facilitated a comprehensive recording of diagnoses by reviewing prescriptions. To prevent duplication, each of the patients received a unique identification number, and those previously interviewed in the current study were excluded later. Exclusions from the study comprised patients unable to participate, those with insufficient cognition, and individuals with debilitating conditions who were unwilling to take part. Four public health professionals with previous experience in multimorbidity data collection conducted the interviews. They were proficient in the local language and skilled in quantitative interviews.

The study received ethical approval from the Institutional Ethics Committee of ICMR-Regional Medical Research Centre, Bhubaneswar (ICMR-RMRCBB/IHEC-2021/93 Date 30/12/2021). Written informed consent was acquired from all participants, with measures in place to guarantee patient confidentiality and anonymity. When patients could not provide necessary information, caregivers were approached for relevant details. Additionally, the hospital and outpatient department’s approval was secured before initiating the data collection process.

### Statistical analysis

The demographic attributes of the patients were depicted through descriptive statistics, specifically frequency and percentage. Recognizing the importance of age as a predictive factor for multimorbidity, as well as its impact on associated health outcomes and HRQoL, we chose to conduct all analyses with age stratification—categorizing individuals into three groups: 18–39 years, 40–59 years, and 60 years and above. Individuals aged 60 and above are officially classified as older people or senior citizens, according to the 1999 National Policy for Older People (20). Disease conditions with a prevalence exceeding 10 percent, such as Hypertension (HT), Diabetes (DB), Acid peptic disease (APD), Arthritis (AT), Chronic respiratory disease (CRD), and Stroke/paralysis (SP), were selected for the calculation of dyads and triads.

Data gathered by the EQ-5D-5L from participants undergo conversion into a health utility index, offering a quantitative assessment of the overall health state. The EQ-5D-5L score, derived from the five questions, spans a theoretical range from −0.923 (indicating a state worse than death) to 1 (representing the best possible health state). The utility values for all 3,125 conceivable health states were sourced from Jyani et al. (16) within the Indian context. The average utility score and standard deviation were computed for each disease condition. Likewise, the mean utility score with standard deviation for multimorbidity conditions—ranging from one to four, and four and above—as well as diverse combinations of dyad and triad among different age groups, was determined. For dyad and triad scenarios, conditions with at least five or more occurrences were taken into account for the calculation of the mean utility score.

In order to explore factors associated with HRQoL, the univariate association between independent variables and HRQoL measures (utility score) was examined using ordinary least squares regression. Additionally, a hierarchical multiple regression analysis was then conducted to determine the relationship between demographic variables, clinical outcomes (multimorbidity conditions) and lifestyle variables (physical activity – exercise) with utility score. In step 1, a linear regression model was constructed to evaluate the relationship between utility score (dependent variables) and demographic variables such as age, sex, residence, education, ethnicity, marital status and occupations. This approach allowed for the assessment of the independent effect of each demographic factor on utility scores while controlling for others. In step 2, the analysis was extended to include clinical outcomes (multimorbidity conditions) and lifestyle variables (physical activity – exercise) were added to the model. A multivariable linear regression model was used to examine the combined influence of these factors on utility scores, with all relevant variables entered simultaneously. The significance level was set at *p* < 0.05. Furthermore, using the distribution-based method, the standard deviation of the utility scores was calculated, and the minimally important difference (MID) was estimated. Statistical analyses were conducted using STATA 16 (Stata Corp, College Station, TX, USA).

## Results

The study included 906 participants, with 276 in the 18–39 age group, 398 in the 40–59 age group, and 232 in the 60 and above age group. In the participant group, 49% were female, 50% were male, and approximately 1% identified as third-gender. Approximately 59% reside in rural areas, 40% belong to the general caste, and 51% have completed high school/senior secondary education. Approximately 39% lacked health insurance coverage, and the mean monthly healthcare expenditure was 2,581 INR (Indian Rupees). Table 1 provides an in-depth overview of the participants’ characteristics.

**Table 1:** Characteristic of study participants by age group.

Participants characteristics	Age groups (years)			
	Total (*N* = 906) *n* (%)	18–39 years *n* = (276) *n* (%)	40–59 years (*n* = 398) *n* (%)	60 + years (*n* = 232) *n* (%)
Gender				
Female	447 (49.3)	144 (52.2)	203 (51.0)	100 (43.1)
Male	454 (50.1)	128 (46.4)	194 (48.7)	132 (56.9)
Third gender	5 (0.6)	4 (1.4)	1 (0.3)	0
Residence				
Urban residential	303 (33.4)	96 (34.8)	117 (29.4)	90 (38.8)
Urban slum	19 (2.1)	8 (2.9)	5 (1.3)	6 (2.6)
Peri-urban	53 (5.9)	12 (4.3)	26 (6.5)	15 (6.5)
Rural	531 (58.6)	160 (58.0)	250 (62.8)	121 (52.1)
Caste				
Scheduled tribe	92 (10.2)	25 (9.1)	44 (11.1)	23 (9.9)
Schedule caste	126 (13.9)	44 (15.9)	57 (14.3)	25 (10.8)
Other Backward Class	320 (35.3)	100 (36.2)	146 (36.7)	74 (31.9)
General	368 (40.6)	107 (38.8)	151 (37.9)	110 (47.4)
Marital status				
With partner	747 (82.5)	205 (74.3)	367 (92.2)	175 (75.4)
Without partner	159 (17.5)	71 (25.7)	31 (7.8)	57 (24.6)
Education				
No formal schooling	62 (6.8)	0	25 (6.3)	37 (16.0)
Up to primary	121 (13.4)	12 (4.3)	71 (17.8)	38 (16.4)
Up to high/senior secondary	461 (50.9)	154 (55.8)	203 (51.0)	104 (44.8)
Graduation and above	262 (28.9)	110 (39.9)	99 (24.9)	53 (22.8)
Occupation				
Currently working	294 (32.4)	117 (42.4)	152 (38.2)	25 (10.8)
Currently not working	299 (33.0)	50 (18.1)	98 (24.6)	151 (65.1)
Never worked	313 (34.6)	109 (39.5)	148 (37.2)	56 (24.1)
Health insurance				
Public scheme	416 (45.9)	118 (42.7)	201 (50.5)	97 (41.8)
Private scheme	140 (15.5)	41 (14.9)	53 (13.3)	46 (19.8)
No health insurance	350 (38.6)	117 (42.4)	144 (36.2)	89 (38.4)
Gross family expenditure per month in INR, mean (SD)	11507.17 (5591.66)	11851.45 (5201.72)	11546.73 (5578.18)	11029.74 (6036.01)
Monthly expenditure in healthcare (INR), mean (SD)	2580.91 (3897.13)	2229.71 (1805.16)	2515.58 (3991.09)	3110.78 (5273.50)

Table 2 details the reported conditions, encompassing a total of 38, with the six most frequently mentioned being hypertension (41%, mean utility score 0.260), diabetes (34%, mean utility score 0.4), acid peptic disease (15%, mean utility score 0.635), arthritis (14%, mean utility score 0.447), chronic respiratory disease (13%, mean utility score 0.493), and stroke/paralysis (12%, −0.349). Notably, conditions such as schizophrenia/unipolar/bipolar disorder (*n* = 3, mean utility score −0.852), stroke/paralysis (*n* = 113, mean utility score −0.349), parkinsonism (*n* = 12, mean utility score −0.204), disability/deformity (*n* = 10, mean utility score −0.111), and dementia/Alzheimer (*n* = 13, mean utility score −0.027) exhibited negative utility scores. Further specifics on disease-specific mean utility scores are presented in Table 2.

**Table 2:** Disease-specific mean utility score.

Conditions	Frequency	Percentage	Mean utility score (SD)
Hypertension	371	41	0.260 (0.646)
Diabetes	306	34	0.400 (0.575)
Acid peptic disease	136	15	0.635 (0.419)
Arthritis	128	14	0.447 (0.472)
Chronic respiratory disease	122	13	0.493 (0.556)
Stroke/paralysis	113	12	−0.349 (0.530)
Thyroid disorder	87	10	0.533 (0.564)
Liver disorder/pancreatitis	87	10	0.549 (0.475)
Chronic kidney disease	70	8	0.409 (0.595)
Chronic back ache	69	8	0.374 (0.512)
Heart disease	55	6	0.397 (0.640)
Migraine	49	5	0.655 (0.308)
Vertigo	44	5	0.575 (0.500)
Chronic constipation	42	5	0.325 (0.587)
Hemoglobinopathy/anaemia	41	5	0.425 (0.530)
Alcohol substance abuse	41	5	0.322 (0.506)
Visual difficulty	39	4	0.722 (0.325)
Hypercholesterolemia	38	4	0.414 (0.609)
Irritable bowel syndrome	37	4	0.365 (0.665)
Piles	35	4	0.720 (0.252)
Sleep disorder	28	3	0.367 (0.622)
Oral conditions	22	2	0.848 (0.101)
Prostatic condition	21	2	0.465 (0.524)
Hearing impairment	17	2	0.279 (0.689)
Chronic nonhealing wound	16	2	0.355 (0.540)
Depression	13	0.01	0.162 (0.572)
Dementia/Alzheimer	13	0.01	−0.027 (0.781)
Parkinsonism	12	0.01	−0.204 (0.569)
Chronic Rhinitis	11	0.01	0.873 (0.096)
Disability/deformity	10	0.01	−0.111 (0.656)
Filariasis	8	0.01	0.185 (0.618)
Tuberculosis	6	0.01	0.363 (0.767)
Eczema	6	0.01	0.502 (0.619)
Cancer	6	0.01	0.357 (0.706)
Psoriasis	5	0.01	0.487 (0.729)
Epilepsy	5	0.01	0.788 (0.225)
Schizophrenia/unipolar/bipolar disorder	3	0.003	−0.852 (0.122)
HIV	1	0.001	0.954

The comprehensive mean utility scores for multimorbidity conditions across different age groups are outlined in Table 3. Of the participants, 19% exhibited one condition with a utility score of 0.677, 45% presented with two conditions, registering a mean utility score of 0.577, 22% had three conditions with a mean utility score of 0.354, and 14% had four or more conditions, reflecting a mean utility score of 0.098 (Table 3). It was noted that across all age groups, as the number of conditions increased, the utility score exhibited a decline. Additionally, the overall utility score in the older age group was found to be lower compared to other age groups. Specifically, within the older age group, when the number of conditions reached four or more, the utility score was recorded as −0.027.

**Table 3:** Mean utility score of multimorbidity conditions among various age groups.

Conditions	All age groups (*N* = 906)		18–39 years (*N* = 276)		40–59 years (*N* = 398)		60 + years (*N* = 232)	
	*n* (%)	Utility score mean (SD)	*n* (%)	Utility score mean (SD)	*n* (%)	Utility score mean (SD)	*n* (%)	Utility score mean (SD)
One	170 (19)	0.677 (0.42)	101 (37)	0.738 (0.31)	50 (13)	0.705 (0.44)	19 (8)	0.279 (0.63)
Two	408 (45)	0.577 (0.49)	136 (49)	0.624 (0.43)	173 (43)	0.625 (0.45)	99 (43)	0.430 (0.59)
Three	203 (22)	0.354 (0.58)	32 (12)	0.358 (0.58)	111 (28)	0.398 (0.53)	60 (26)	0.270 (0.65)
Four and above	125 (14)	0.098 (0.65)	7 (2)	0.608 (0.23)	64 (16)	0.148 (0.65)	54 (23)	−0.027 (0.64)

**Figure 1:**
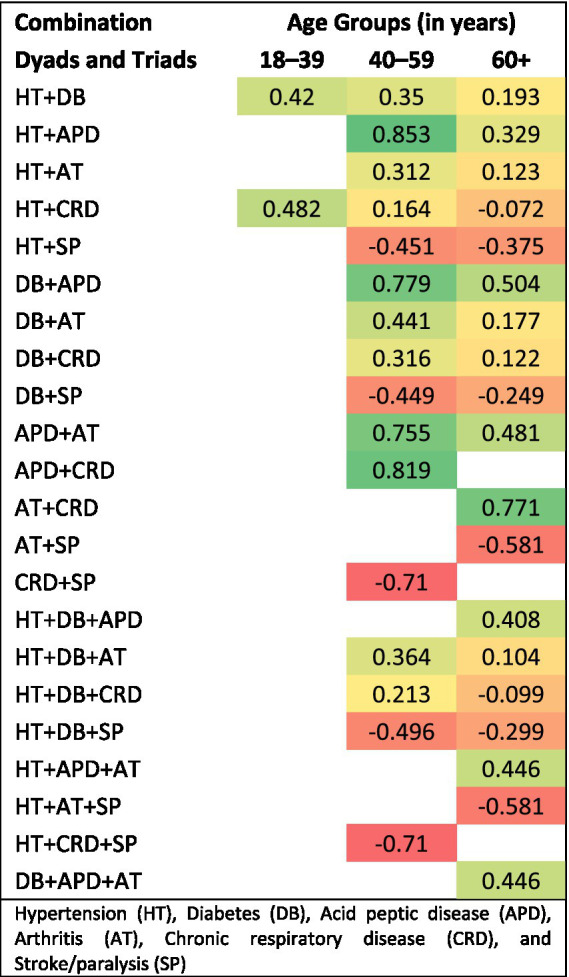
Heatmaps on the influence of dyads and triads on utility scores by age group.

Table 4 presents the mean utility scores of multimorbidity, considering various combinations (dyad and triad) of conditions across different age groups. Within dyad conditions, the pairing of stroke/paralysis with other conditions like hypertension (mean utility score −0.403), diabetes (mean utility score −0.338), acid peptic disease (mean utility score −0.091), arthritis (mean utility score −0.519), and chronic respiratory disease (mean utility score −0.710) resulted in a negative value. Likewise, combining diabetes with chronic respiratory disease yielded a negative value, with a mean utility score of −0.636. Similarly, in triad conditions, combining stroke/paralysis with other health conditions led to a negative value. Within both dyad and triad combinations, the mean utility score for Quality of Life in older age groups was relatively lower than in other age groups. Figure 1 shows the heatmaps on the influence of dyads and triads on utility scores by age group.

**Table 4:** Mean utility score of multimorbidity with various combinations (dyad and triad) of conditions among various age groups.

Multimorbidity		All age groups (*N* = 906)		18–39 years (*N* = 276)		40–59 years (*N* = 398)		60 + years (*N* = 232)	
Conditions	Combination	*n* (%)	Mean utility score (SD)	*n* (%)	Mean utility score (SD)	*n* (%)	Mean utility score (SD)	*n* (%)	Mean utility score (SD)
Dyad	HT + DB	214 (23.6)	0.295 (0.607)	13 (4.7)	0.42 (0.645)	120 (30.2)	0.350 (0.576)	81 (34.9)	0.193 (0.636)
	HT + APD	23 (2.5)	0.528 (0.522)	1 (0.36)	–	8 (2.0)	0.853 (0.066)	14 (6.0)	0.329 (0.590)
	HT + AT	62 (6.8)	0.242 (0.528)	3 (1.1)	–	31 (7.8)	0.312 (0.427)	28 (12.1)	0.123 (0.621)
	HT + CRD	45 (5.0)	0.154 (0.667)	6 (2.2)	0.482 (0.591)	29 (7.3)	0.164 (0.642)	10 (4.3)	−0.072 (0.756)
	HT + SP	97 (10.7)	−0.403 (0.492)	4 (1.2)	–	42 (10.6)	−0.451 (0.489)	51 (23.0)	−0.375 (0.478)
	DB + APD	26 (2.9)	0.609 (0.351)	1 (0.4)	–	12 (3.0)	0.779 (0.167)	13 (5.6)	0.504 (0.388)
	DB + AT	60 (6.6)	0.321 (0.527)	1 (0.4)	–	31 (7.8)	0.441 (0.380)	28 (12.1)	0.177 (0.637)
	DB + CRD	30 (3.3)	−0.636 (0.405)	3 (1.1)	–	19 (4.8)	0.316 (0.509)	8 (3.5)	0.122 (0.697)
	DB + SP	49 (5.4)	−0.338 (0.508)	3 (1.1)	–	25 (6.3)	−0.449 (0.459)	21 (9.0)	−0.249 (0.519)
	APD + AT	14 (1.6)	0.602 (0.259)	1 (0.4)	–	5 (1.3)	0.755 (0.139)	8 (3.5)	0.481 (0.273)
	APD + CRD	12 (1.3)	0.537 (0.520)	4 (1.5)	–	5 (1.3)	0.819 (0.108)	3 (1.2)	–
	APD + SP	2 (0.2)	−0.091 (1.177)	0	–	1	–	1	–
	AT+CRD	12 (1.3)	0.732 (0.152)	2 (0.7)	–	3 (0.7)	–	7 (3.0)	0.771 (0.097)
	AT+SP	9 (1.0)	−0.519 (0.244)	0	–	4 (1.0)	–	5 (2.2)	−0.581 (0.312)
	CRD + SP	5 (0.5)	−0.710 (0.292)	0	–	5 (1.3)	−0.710 (0.292)	0	–
Triad	HT + DB + APD	13 (1.4)	0.551 (0.408)	0	–	4 (1.0)	–	9 (3.9)	0.408 (0.415)
	HT + DB + AT	46 (5.1)	0.223 (0.537)	0	–	21 (5.3)	0.364 (0.356)	25 (10.8)	0.104 (0.636)
	HT + DB + CRD	20 (2.2)	0.184 (0.612)	2 (0.7)	–	13 (3.3)	0.213 (0.560)	5 (2.2)	−0.099 (0.733)
	HT + DB + SP	46 (5.1)	−0.380 (0.476)	3 (1.1)	–	23 (5.8)	−0.496 (0.419)	20 (8.6)	−0.299 (0.477)
	HT + APD + AT	7 (0.8)	0.504 (0.270)	0	–	1 (0.3)	–	6 (2.6)	0.446 (0.244)
	HT + APD + CRD	1 (0.1)	0.614	0	–	0	–	1	–
	HT + APD + SP	2 (0.2)	−0.091 (1.177)	0	–	1	–	1	–
	HT + AT+CRD	4 (0.4)	0.621 (0.200)	1	–	0	–	3 (1.2)	–
	HT + AT+SP	8 (0.9)	−0.527 (0.260)	0	–	3 (0.7)	–	5 (2.2)	−0.581 (0.312)
	HT + CRD + SP	5 (0.5)	−0.710 (0.292)	0	–	5 (1.3)	−0.710 (0.292)	0	–
	DB + APD + AT	8 (0.9)	0.524 (0.256)	0	–	2 (0.5)	–	6 (2.6)	0.446 (0.244)
	DB + APD + CRD	1 (0.1)	0.614	0	–	0	–	1	–
	DB + APD + SP	0	-	0	–	0	–	0	–
	DB + AT+CRD	4 (0.4)	0.714 (0.071)	0	–	1	–	3 (1.3)	–
	DB + AT+SP	6 (0.6)	−0.561 (0.284)	0	–	2	–	4 (1.7)	–
	DB + CRD + SP	2 (0.2)	−0.636 (0.405)	0	–	2	–	0	–
	APD + AT+CRD	3 (0.3)	0.766 (0.138)	1	–	1	–	1	–
	APD + AT+SP	0	–	0	–	0	–	0	–
	APD + CRD + SP	0	–	0	–	0	–	0	–
	AT+CRD + SP	0	–	0	–	0	–	0	–

Hypertension (HT); diabetes (DB); acid peptic disease (APD); arthritis (AT); chronic respiratory disease (CRD); and stroke/paralysis (SP).

The univariate analysis results (Table 5) showed that younger groups (18–39 and 40–59) have significantly higher utility scores (better HRQoL) compared to the ≥60 reference group. The 18–39 age group shows the strongest positive association. Urban slum residents and urban residents generally have higher utility scores compared to rural residents, with urban slum residents showing a significant difference. Higher education levels (up to high/senior secondary and graduation) are significantly associated with higher utility scores. Having a partner was significantly associated with higher utility scores. Individuals who were currently employed had higher utility scores. Sometimes or regularly exercising was associated with higher utility scores. More health conditions were associated with lower utility scores. Males and females, as well as ethnicity, did not significantly differ in their utility scores.

**Table 5:** Regression analysis results with utility score as the outcome variable.

Variables	Univariate analysis		Multivariate analysis			
	*β*-coeff (95% CI)	*p*-value	Model 1		Model 2	
			*β*-coeff (95% CI)	*p*-value	*β*-coeff (95% CI)	*p*-value
Age group (in years)						
60 and above	Ref		Ref		Ref	
40–59	0.225 (0.137, 0.312)	0.001	0.144 (0.055, 0.234)	0.002	0.118 (0.035, 0.202)	0.006
18–39	0.364 (0.270, 0.459)	0.001	0.262 (0.162, 0.363)	0.001	0.152 (0.053, 0.251)	0.003
Sex						
Female	Ref		Ref		Ref	
Male	0.015 (−0.058, 0.087)	0.684	−0.052 (−0.126, 0.022)	0.165	−0.105 (−0.176, −0.035)	0.003
Transgender	0.261 (−0.229, 0.752)	0.296	0.065 (−0.408, 0.538)	0.788	0.106 (−0.338, 0.550)	0.64
Residence						
Rural	Ref		Ref		Ref	
Peri-urban	0.021 (−0.133, 0.175)	0.79	−0.045 (−0.194, 0.103)	0.549	−0.008	0.91
Urban slum	0.281 (0.030, 0.531)	0.028	0.310 (0.060, 0.559)	0.015	0.237	0.048
Urban residence	0.215 (0.137, 0.292)	0.001	0.095 (0.006, 0.184)	0.037	0.095	0.027
Education						
No formal schooling	Ref		Ref		Ref	
Up to primary	0.216 (0.053, 0.378)	0.009	0.105 (−0.054, 0.265)	0.194	0.083 (−0.066, 0.233)	0.276
Up to high/senior secondary	0.457 (0.316, 0.597)	0.001	0.257 (0.111, 0.403)	0.001	0.180 (0.041, 0.318)	0.011
Graduation	0.619 (0.473, 0.766)	0.001	0.339 (0.170, 0.508)	0.001	0.195 (0.034, 0.357)	0.018
Ethnicity						
Scheduled Tribe	Ref		Ref		Ref	
Schedule Caste	−0.001 (−0.149, 0.147)	0.99	−0.017 (−0.155, 0.122)	0.814	0.010 (−0.120, 0.140)	0.881
Other Backwards Class	0.098 (−0.030, 0.226)	0.134	0.045 (−0.076, 0.167)	0.462	0.072 (−0.042, 0.186)	0.215
General	0.187 (0.061, 0.313)	0.004	0.120 (−0.003, 0.244)	0.057	0.124 (0.007, 0.241)	0.037
Marital status						
Without partner	Ref		Ref		Ref	
With partner	0.262 (0.168, 0.356)	0.001	0.255 (0.162, 0.347)	0.001	0.208 (0.120, 0.295)	0.001
Occupation						
Currently not working	Ref		Ref		Ref	
Currently working	0.257 (0.182, 0.333)	0.001	0.116 (0.032, 0.201)	0.007	0.112 (0.033, 0.192)	0.006
Physical activity (exercise)						
Never	Ref				Ref	
Sometimes	0.356 (0.281, 0.431)	0.001			0.243 (0.169, 0.317)	0.001
Regularly	0.416 (0.311, 0.522)	0.001			0.249 (0.142, 0.356)	0.001
Number of disease conditions						
One condition	Ref				Ref	
Two conditions	0.255 (0.139, 0.372)	0.001			0.211 (0.103, 0.318)	0.001
Three conditions	0.479 (0.374, 0.584)	0.001			0.364 (0.263, 0.464)	0.001
Four or more conditions	0.579 (0.457, 0.700)	0.001			0.418 (0.298, 0.537)	0.001
R-squared			0.180		0.285	
Adjusted R-squared			0.166		0.269	
*F*			13.03*		17.68*	

*p-value < 0.05.

When adjusted for basic demographics (model 1), such as age, residence, education, marital status, occupation, physical activity, and number of conditions, remain significantly associated with utility scores (Table 5). When the fully adjusted model, including all variables (model 2), includes physical activity and multimorbidity conditions, younger age groups still had higher utility scores, urban residents continue to show higher utility scores, and higher education remains positively associated. Having a partner continues to predict higher utility scores. Regular and sometimes exercising had been linked with better utility scores. However, more disease conditions reduce utility scores (Table 5). The full model explains about 27% (adjusted *R*^2^ ≈ 0.27) of the variation in utility scores, which was moderate. The *F*-test indicates the models were statistically significant overall.

Across the sample, utility scores varied widely, with an overall mean of approximately 0.48, reflecting moderate health-related quality of life. Participants with multiple conditions tended to have lower average utility scores, while those with only one condition reported higher scores. The variability was substantial, with standard deviations ranging from about 0.42–0.65, and scores spanning from negative values (worse than death) up to perfect health (Table 6).

**Table 6:** Overall summary of utility scores and minimal important differences (MID).

Number of disease conditions	*N*	Mean utility score	Standard deviation	Range	MID (0.5 × SD)	Clinical significance
Overall	906	0.48	0.56	−0.923 to 1	0.28	A change of ≥0.28 points in utility score is clinically meaningful, indicating a noticeable difference in HRQoL
Participants with 4+ conditions	125	0.098	0.65	−0.923 to 1	0.32	Improvements or declines of ≥0.32 are meaningful, with this group reporting the lowest HRQoL
Participants with 3 conditions	203	0.35	0.58	−0.923 to 1	0.29	Changes ≥0.29 are significant; moderate HRQoL
Participants with 2 conditions	408	0.58	0.49	−0.923 to 1	0.25	Changes ≥0.25 are perceptible; relatively good health status
Participants with 1 condition	170	0.68	0.42	−0.923 to 1	0.21	Changes ≥0.21 are meaningful; higher average utility, indicating better health

## Discussion

The study evaluated the HRQoL of multimorbid patients using the EQ-5D-5L value set for India. It identified prevalent health conditions such as hypertension, diabetes, and stroke, revealing a significant decline in mean utility scores as the number of conditions increased. Specifically, utility scores declined from 0.677 for one condition to 0.098 for four or more conditions, with older adults particularly affected. Negative utility scores were associated with severe conditions like schizophrenia and stroke. The study found that younger age, urban living, higher education, marital status, regular physical activity, and fewer health conditions correlated with higher utility scores. The minimum important difference for perceived meaningful change in utility scores ranged from 0.21 to 0.32 points, indicating that more significant changes are necessary for those with multiple conditions.

Understanding the patterns of common health conditions is critical for healthcare professionals and policymakers in developing targeted interventions. Recognizing the specific conditions that are common in a population allows healthcare professionals to tailor their approaches to early detection, diagnosis, and treatment. This knowledge enables the development of specialised care plans and interventions that address the distinct challenges presented by each common health condition. As a result, the HRQoL measurement is critical for understanding how chronic conditions, multimorbidity, and polypharmacy affect patients’ health. Studies such as Bhadhuri et al. (21) and Wong et al. (22) emphasised the importance of using standardised instruments such as the EQ-5D-3L and EQ-5D-5L to assess self-reported health status among older patients with significant multimorbidity and polypharmacy (21–23). These studies highlight the need for comprehensive evaluation tools that can capture the complexities of health conditions and their impact on HRQoL, particularly in vulnerable populations. Furthermore, Peters et al.’s study sheds light on the impact of multimorbidity on HRQoL among primary care patients, emphasising the importance of self-efficacy in managing multiple health conditions (6, 24). Understanding the relationship between self-efficacy and HRQoL is critical for designing interventions that assist patients in dealing with their health issues and improving their overall wellbeing.

To improve responsiveness to the individual needs and experiences of patients with multiple health conditions, healthcare systems must take a holistic, patient-centred approach. Integrated care models, which encourage collaboration among various healthcare professionals, are critical for addressing the interconnected complexities of multimorbidity. Community collaboration addresses social determinants of health, and continuous monitoring and feedback systems enable timely adjustments to care plans. Previous research has highlighted the high healthcare costs associated with multimorbidity and its impact on HRQoL, particularly after hospitalisation (25–27). These findings emphasise the importance of integrated and patient-centred care models that address the complex needs of multimorbid patients while also considering the economic implications of their healthcare management. Furthermore, previous research has shed light on the catalogue of chronic conditions and psychosocial factors associated with HRQoL, contributing to our understanding of the multifaceted nature of HRQoL assessment in chronic disease patients (28–31). The findings highlight the importance of taking a comprehensive approach to assessing HRQoL in patients with multimorbidity, taking into account both clinical and psychosocial factors, to better inform healthcare interventions and outcomes.

Individuals with multimorbidity are more likely to use health-care services, require complex medications, and have polypharmacy than those without. As a result, recognising and addressing multimorbidity is critical in shaping the design of effective healthcare systems. Previous studies relied on value sets from Thailand and the United Kingdom because there was no India-specific EQ-5D-5L value set available. As a result, developing an India-specific EQ-5D-5L value set became critical to ensuring more transparent and consistent decision-making (16). This analysis improves understanding of individuals’ health-related quality of life, providing useful insights for healthcare professionals, researchers, and policymakers (32, 33). It makes it easier to assess the effects of interventions, allows for more informed decision-making, and contributes to population wellbeing.

This study examines the impact of identified health conditions on quality of life, particularly those with low utility scores, as well as the difficulties and implications of managing multiple conditions. The study expands on age disparities, focusing on lower mean utility scores in the older age group with multiple conditions, and investigates potential causes and implications for tailored healthcare strategies (31). The importance of holistic healthcare approaches is emphasised, recognising the complex interplay of multiple health conditions and proposing strategies for healthcare systems to better support individuals with multimorbidity, particularly in ageing populations. Policymakers can use this information to better allocate resources, design public health campaigns, and implement policies that address the identified health concerns. Furthermore, understanding these patterns helps predict future healthcare needs and design preventive strategies. This targeted approach not only optimises healthcare resource allocation but also improves intervention effectiveness, resulting in better health outcomes for both individuals and communities. In essence, a nuanced understanding of common health conditions enables healthcare professionals and policymakers to implement strategic and effective measures that address the specific health needs of the population they serve.

The results emphasise that conditions such as stroke/paralysis and schizophrenia/unipolar/bipolar disorder are associated with significantly negative utility scores, which suggests a significant decrease in quality of life. In contrast, diseases such as acid peptic disease and diabetes, despite their prevalence, have relatively higher mean utility scores, which implies a less significant impact on HRQoL (5). An analysis that is more nuanced and investigates the impact of specific disease combinations, such as stroke with hypertension or arthritis with chronic respiratory disease, on the overall decline in utility scores, would offer a more comprehensive understanding of the relative burden of various conditions. Furthermore, the interpretation of these results would be strengthened by an understanding of the reasons why certain conditions have a more profound impact, whether it be due to the severity of symptoms, disability, or psychological effects. Targeted interventions to enhance HRQoL among patients with multimorbidity would be informed by such an exhaustive investigation, which would also contextualise the quantitative findings.

The results have substantial implications for the geriatric populations of India and other LMICs. As the prevalence of multimorbidity increases, older individuals experience significant declines in HRQoL, with the mean utility decreasing by nearly four times (from 0.677 to 0.098). The low scores were linked to severe conditions, including schizophrenia and stroke, underscoring the necessity of targeted integrative interventions in the older adults population to enhance their quality of life. Moreover, the estimation of QALYs at the population level will be facilitated by quantified disease-specific HRQoL, which will be useful for cost-effectiveness research and the allocation of resources for any rehabilitation programs, assistive devices, and home-based care among the geriatric population in India. Additionally, early screening and care for anxiety and depression among older populations may enhance QALYs and their opportunities for social engagement.

### Implications for policy and practices

- Promoting lifestyle modifications, recognizing multimorbidity’s social and psychological implications, and investing in community-based support programs, mental health services, and medication subsidies can improve access and affordability for patients.
- Nurses should promote lifestyle modifications such as regular physical activity and health education among patients, particularly in younger populations. Enhancing access to preventive and early diagnostic services, especially in rural and underserved areas. In order to address the holistic needs of patients, nurses should support community-based programs and mental health services.
- This study recommends clinicians for prioritizing integrated care models for managing multiple conditions through comprehensive care approaches. Incorporate utility score assessments into clinical evaluations to enhance comprehension of patient requirements. Recognizing the psychological effects of multimorbidity, including stress, anxiety, and depression, and integrating psychosocial support into care.
- Policymakers should improve access to preventive services, invest in community-based support programs, and subsidize essential medications. Addressing social determinants of health and supporting research on sociodemographic factors can improve healthcare access and quality of life.

### Limitations of the study

The study faces limitations regarding its sampling method and sample size, which may affect the generalizability of its findings on India’s multimorbid patient population. Although efforts were made to include participants from both private and public healthcare sectors, the recruitment from two tertiary hospitals in a single state may not represent the diversity of multimorbid patients across the country. The sample size, calculated based on relevant parameters, was smaller than intended, particularly in the private sector, hindering comparisons of HRQoL between public and private facilities. Moreover, given the observational study design and the sampling strategy employed, several potential biases may influence the study findings. Selection bias could arise due to the inclusion of only patients attending the medicine outpatient departments on designated days, potentially excluding individuals with different health-seeking behaviors or those unable to visit the hospital, particularly the severely ill, disabled, or those from remote areas, which may limit the generalizability of the results. The patients with cognitive impairments or debilitating conditions were excluded, which could underestimate the prevalence or impact of multimorbidity in the broader patient population.

Additionally, the sample was insufficient to analyze disease combinations by burden and their impact on quality of life, potentially introducing bias and affecting precision. Exclusion criteria, such as cognitive ability and refusal to participate, may have omitted valuable insights. Although the findings have demonstrated that an increase in the number of diseases results in a decrease in the health utility score, however, the relationship between specific diseases and their severity of impact on HRQoL is not thoroughly examined. These limitations highlight the need for further research with larger, more diverse samples to enhance the robustness and applicability of the findings. The study reports disease-specific utility scores and explores combinations of conditions, but it does not conduct an in-depth analysis of the individual impact of each disease on HRQoL within the regression models. These limits understanding of how specific conditions, such as depression or diabetes, independently influence quality of life, which could be addressed in future analyses for more nuanced insights.

## Conclusion

The findings reveal that multimorbidity significantly reduces HRQoL, particularly in older adults with multiple health conditions. Moreover, conditions like stroke, parkinsonism, and dementia notably worsen utility scores, especially when combined. The findings stress the necessity for comprehensive healthcare strategies aimed at improving wellbeing in complex medical scenarios. Furthermore, context-specific minimal important difference thresholds are crucial for interpreting utility scores; smaller changes are significant for those with fewer conditions, while larger shifts are needed for those with multiple ailments. This understanding is vital for clinicians and researchers in assessing interventions and monitoring disease progression effectively.

## Data Availability

The raw data supporting the conclusions of this article will be made available by the authors, without undue reservation.
